# Slowly Delivered Icariin/Allogeneic Bone Marrow-Derived Mesenchymal Stem Cells to Promote the Healing of Calvarial Critical-Size Bone Defects

**DOI:** 10.1155/2016/1416047

**Published:** 2016-09-15

**Authors:** Tianlin Liu, Xin Zhang, Yuan Luo, Yuanliang Huang, Gang Wu

**Affiliations:** ^1^Laboratory of Oral Biomedical Science and Translational Medicine, Department of Oral and Maxillofacial Surgery, School of Stomatology, Tongji University, Shanghai, China; ^2^Department of Stomatology, Shanghai East Hospital Affiliated with Tongji University, Jimo Road No. 150, Shanghai 200120, China; ^3^Stomatology Hospital Affiliated with Zhejiang University College of Medicine, Hangzhou, China; ^4^Shanghai Stomatosis Prevention and Treatment Center, Shanghai, China; ^5^Department of Oral Implantology and Prosthetic Dentistry, Academic Center for Dentistry Amsterdam (ACTA), MOVE Research Institute, University of Amsterdam and VU University Amsterdam, Gustav Mahlerlaan 3004, 1081 LA Amsterdam, Netherlands

## Abstract

Bone tissue engineering technique is a promising strategy to repair large-volume bone defects. In this study, we developed a 3-dimensional construct by combining icariin (a small-molecule Chinese medicine), allogeneic bone marrow-derived mesenchymal stem cells (BMSCs), and a siliceous mesostructured cellular foams-poly(3-hydroxybutyrate-co-3-hydroxyhexanoate) (SMC-PHBHHx) composite scaffold. We hypothesized that the slowly released icariin could significantly promote the efficacy of SMC-PHBHHx/allogeneic BMSCs for repairing critical-size bone defects in rats. In in vitro cellular experiments, icariin at optimal concentration (10^−6^ mol/L) could significantly upregulate the osteogenesis- and angiogenesis-related genes and proteins, such as Runx2, ALP, osteocalcin, vascular endothelial growth factors, and fibroblast growth factors, as well as the mineralization of BMSCs. Icariin that was adsorbed onto the SMC-PHBHHx scaffold showed a slow release profile within a 2-week monitoring span. Eight weeks after implantation in calvarial critical-size bone defects, the constructs with icariin were associated with significantly higher bone volume density, trabecular thickness, trabecular number, and significantly lower trabecular separation than the constructs without icariin. Histomorphometric analysis showed that icariin was also associated with a significantly higher density of newly formed blood vessels. These data suggested a promising application potential of the icariin/SMC-PHBHHx/allogeneic BMSCs constructs for repairing large-volume bone defects in clinic.

## 1. Introduction

Large-volume bone defects may result from congenital nonunion, trauma, inflammation, and clinical treatments such as osteosarcoma-resection. The osseous repair of large-volume bone defects is still a challenge in the fields of orthopedics, maxillofacial surgery, and dental implantology. Although autologous bone grafts are routinely adopted to treat large-volume bone defects, the disadvantages of these grafts (e.g., limited quantity, donor site morbidity) have engendered tremendous efforts to develop alternatives [1], among which bone tissue engineering technique is highly promising [2, 3]. Bone tissue engineering technique is an interdisciplinary science that applies the principles of biology and engineering to develop viable substitutes for restoring, maintaining, or improving the function of bone tissue. A good combination of osteogenic cells, osteopromotive drugs, and osteoconductive biomaterials is critical for the success of this technique.

The accumulation, proliferation, and osteogenic differentiation of multipotent mesenchymal stem cells (MSCs) are indispensable for a proper fracture healing [4]. Thereby, bone marrow-derived MSCs- (BMSCs-) based strategies are introduced to promote the healing of bone fractures and other bone metabolic diseases in clinics [5]. Their ability to modulate immune responses enables the application of allogeneic BMSCs without a substantial risk of immune rejection [5]. Continuous attempts have already been performed in preclinical models to apply allogeneic BMSCs in promoting the repair of bone defects [6]. In segmental critical-size bone defects, allogeneic BMSCs could significantly promote bone regeneration in a comparable level with autologous BMSCs [7]. The application of allogeneic BMSCs is also advantageous over autologous BMSCs due to their timeliness and sufficient availability [8].

Growth factors can be frequently adopted to further enhance and accelerate bone healing process. In the field of bone regeneration, bone morphogenetic proteins (BMPs) are the most important growth factors. Recombinant human BMP-2 and BMP-7 have been proved to significantly promote bone formation both in animal models and in clinical trials [9–11]. However, the effective doses of BMPs for current clinical use are always too high [12, 13], which results in a substantial economic burden to patients and healthcare system. Furthermore, the transiently high dosage may also lead to a series of potential side effects, such as the overstimulation of osteoclastic bone resorption [14], which may compromise its therapeutic effect. For promoting bone repair, one of the viable alternatives can be Chinese medicine, such as icariin. Icariin, a small-molecule drug extracted from a Chinese traditional medicine Herba Epimedii [15], shows a very promising potential. Icariin has been shown to enhance in vitro osteoblastogenesis [16, 17] through the induction of endogenous BMP-2 and nitric oxide (NO) [18, 19]. On the other hand, icariin is also able to reduce osteoclastogenesis through suppressing the signaling of MAPKs/NF-*κ*B (mitogen-activated protein kinase/nuclear factor kappa-light-chain-enhancer of activated B cells) [20] and enhancing the ratio of OPG/RANKL [21]. In addition, icariin can promote angiogenesis, which may further facilitate the repair of large-volume bone defects. In comparison with BMPs, icariin, as a small-molecule drug, can also be easily synthesized, presenting an inexpensive drug to promote bone regeneration.

In the field of bone tissue engineering, an ideal scaffold should have a proper biodegradability and a good biocompatibility to accommodate osteogenic cells. Furthermore, a good slow-delivery capacity is also critical for prolonging the release profile of bioactive agents, thereby maximizing their biological effects. In this study, we adopted a novel composite SMC-PHBHHx (20 : 80) that incorporates biocompatible siliceous mesostructured cellular foams (SMC) [22] into high-toughness and easily moldable poly(3-hydroxybutyrate-co-3-hydroxyhexanoate) (PHBHHx) [23] using a solvent casting and salt-leaching method [24]. The highly porous and well-interconnected structure conferred excellent physicochemical, biological, and drug-release properties on this novel composite scaffold.

Hitherto, it remains unclear whether icariin can promote the efficacy of allogeneic BMSCs-based tissue engineering technique in repairing large-volume bone defects. In this study, we first selected the best concentration of icariin by assessing its effect on promoting the proliferation and early differentiation of allogenic BMSCs. Thereafter, we evaluated the promoting effect of icariin on the osteogenesis- and angiogenesis-related genes and proteins. Finally, we evaluated the efficacy of slowly delivered icariin from the novel SMC-PHBHHx scaffold to treat in vivo calvarial critical-size bone defects in rats through radiographic and histomorphometric analysis. We hypothesized that the slowly released icariin could significantly promote the efficacy of SMC-PHBHHx/allogeneic BMSCs for repairing critical-size bone defects.

## 2. Materials and Methods

### 2.1. In Vitro Cellular Evaluation

#### 2.1.1. Culture of BMSCs

BMSCs in passage 1 were purchased from ATCC and cultured with DMEM (Dulbecco's Modified Eagle Medium, Cyagen, Guangzhou, China) supplemented with 10% fetal bovine serum (FBS), 100 U/mL penicillin, and 100 mg/mL streptomycin at 37°C under the atmosphere of 5% CO_2_ and 100% relative humidity. The passage was carried out when the cells were confluent to 80%. The cells of passage 4 were used in the following studies.

#### 2.1.2. Multiple Differentiation

To identify the multipotency, the allogeneic BMSCs were induced to osteogenic, chondrogenic, and adipogenic differentiation using osteogenic medium, adipogenic medium, and chondrogenic medium (Cyagen, Guangzhou, China), respectively. The medium was replaced every 3 days. The osteogenic, chondrogenic, and adipogenic differentiation were examined at 14 days, 21 days, and 22 days, respectively. The staining was done using an alizarin red staining solution and oil red staining solution (Cyagen, Guangzhou, China) to check osteogenesis and adipogenesis, respectively. In order to check chondrogenesis, the chondrogenic cell pellets were fixed, embedded in paraffin, sectioned, and stained with alcian blue staining solution (Cyagen, Guangzhou, China) to detect the glycosaminoglycans. The images of various differentiations were captured using light microscope (Eclipse Ti-U, Nikon, Tokyo, Japan).

#### 2.1.3. Concentration Selection Test

To identify the optimal concentration of icariin, cell proliferation assay and alkaline phosphatase (ALP) activities were performed with a time-course and dose-dependent setup. Cell proliferation was assessed using the cell counting kit-8 (CCK-8) (Yeasen, Shanghai, China). 2000 BMSCs per well were seeded in 96-well plates. After refreshing the medium 24 h after seeding, 100 *μ*L of culture medium with different concentrations (0, 10^−9^, 10^−8^, 10^−7^, 10^−6^, 10^−5^, and 10^−4^ Mol/L) of icariin (Tauto Biotech, Shanghai, China) was added to each well. Six wells per group were measured. The treatment medium was refreshed every 3 days. After 1-, 3-, 5-, and 7-day incubation, the treatment medium was replaced with 100 *μ*L CCK-8 working solution according to the manufacture's instruction. After a 40 min incubation, the OD (optical density) values were measured at 450 nm. ALP activity and total protein content were measured after the treatment for 1 day, 3 days, 5 days, and 7 days. ALP activity was determined using LabAssay ALP colorimetric assay kit (Wako Pure Chemicals, Osaka, Japan). The total protein content was measured at 570 nm using a commercial BCA Protein Assay kit (Beyotime, Shanghai, China) to normalize the ALP activity.

#### 2.1.4. The Expression of Osteogenesis and Angiogenesis-Related Genes and Proteins

To identify the effect of icariin on osteogenesis-related genes and proteins, BMSCs were cultured in an osteogenic medium as described above. For the angiogenesis-related genes and proteins, BMSCs were cultured in full culture medium without extra inductive agents. The effects of icariin on stimulating the expression of osteogenic and angiogenic genes were examined by quantitative RT-PCR 3 days, 5 days, 7 days, and 14 days after treatment. Total RNA was extracted from the cells using a Trizol Kit (Invitrogen, USA). The cDNA was synthesized from total RNA with a Primescrip™ RT Reagent Kit (Takara Biotechnology, Dalian, China). Real-time polymerase chain reaction (PCR) was performed using 1 *μ*L of cDNA product in a 25 *μ*L reaction volume with Mastercycler® ep realplex Real-Time PCR System (Eppendorf, Germany). In each PCR reaction, SYBR® Premix Ex Taq™ II (Takara Biotechnology), specific primers (Table 1), and 1 *μ*L of cDNA were used according to the manufacturer's instructions. GAPDH was used as housekeeping gene. We calculated the folds of upregulation for each gene of interest using the following formula: 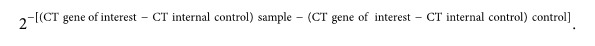
(1)


Western blot analysis was used to assess the expression level of osteogenesis- and angiogenesis-related proteins such as ALP, osteocalcin (OCN), runt-related transcription factor 2 (Runx2), vascular endothelial growth factor (VEGF), and fibroblast growth factors (FGF). After 3 days, 5 days, 7 days, and 14 days, cells lysis was made using M-PER Mammalian Protein Extraction Reagent (ThermoFisher, USA) with a protease and phosphatase inhibitor cocktail (Sigma, USA). Anti-rat primary antibodies were used to detect osteogenesis- and angiogenesis-related proteins ALP, OCN, Runx2, FGF, and VEGF. Horseradish peroxidase-labeled secondary antibodies were then used to label detect the primary antibodies. Images were acquired using darkroom development techniques for chemiluminescence. Image-Pro Plus 6.0 software was adopted to analyze the Integral Optical Density (IOD).

#### 2.1.5. Cell Matrix Mineralization

The effect of 10^−6^ mol/L icariin on the matrix mineralization of BMSCs was examined in osteogenic medium as described in Multilineage Differentiation of Allogeneic BMSCs with DMSO as control. After 14- and 21-day treatments, mineralized nodules were determined by alizarin red staining. Culture plates were photographed using NISElementsF2.20 (Eclipse 80i, Nikon, Tokyo, Japan), and the calcified area was quantified using Image-Pro Plus 6.0 software.

### 2.2. In Vivo Study

#### 2.2.1. Preparation of SMC-PHBHHx Composite

The SMC-PHBHHx composite was fabricated as previously reported [24]. Briefly, to prepare SMC, 1.6 M HCl solution containing 0.53 mM Pluronic P123 (BASF, Frankfurt, Germany) and 0.23 M 1,3,5-triethylbenzene was stirred and kept in 40°C for 60 minutes. Thereafter, tetraethyl orthosilicate was added to reach the final concentration of 0.28 M and reacted for 20 hours at 40°C. The mixture was then subjected to an autoclave at 100°C for 24 hours under static conditions. After cooling at room temperature, the white precipitate was collected, dried, and calcined at 550°C for 6 hours to produce the SMC materials. To prepare SMC/PHBHHx composite, SMC was added to the chloroform containing PHBHHx (20 wt%) to a final concentration of 5 wt% with stirring for 24 hours. After adding NaCl particles with diameters ranging from 300 to 500 *μ*m, the mixture was cast into cylindrical PTFE molds. The samples were then air-dried under flowing air for 24 hours and subsequently vacuum-dried at 40°C for 48 hours. The NaCl particles were then removed by immersing in deionized water for 72 hours with the water replaced every 6 hours. Thereafter, the samples were air-dried for 24 hours and vacuum-dried overnight to obtain the sponge-like scaffolds.

#### 2.2.2. Preparing the Constructs of Icariin/SMC-PHBHHx/Allogeneic BMSCs

To prepare icariin/SMC-PHBHHx composite, we adopted SMC-PHBHHx (20 : 80) discs (5 mm in diameter and 1.5 mm in thickness) and 10^−6^ mol/L icariin suspension in DMEM. 200 *μ*L of either icariin-containing or non-icariin-containing suspension was then adsorbed onto each SMC-PHBHHx disc with a mild shaking for 72 hours. The icariin/SMC-PHBHHx discs were then freeze-dried under sterile condition for 48 hours. The discs were thereafter stored in 4°C for later use. Allogeneic BMSCs were seeded onto either icariin-containing or non-icariin-containing SMC-PHBHHx discs with a mild shaking for 72 hours at 37°C under 5% CO_2_ before implantation.

#### 2.2.3. Characterization of Icariin/SMC-PHBHHx Constructs

*(1) Scanning Electronic Microscope*. Scanning electron microscope (Phenom™ Pro, Eindhoven, The Netherlands) was adopted to reveal the influence of icariin adsorption on the surface morphologies of SMC-PHBHHx composite and BMSCs. For this purpose, either icariin-containing or non-icariin-containing SMC-PHBHHx discs with or without allogeneic BMSCs were mounted on aluminium stubs and sputtered with gold particles.

*(2) Release Kinetics of BSA In Vitro*. The release kinetics of icariin was monitored over a 15-day period in vitro using a high-performance liquid chromatography (BAS PM-80, West Lafayette, IN). Each sample (*n* = 6) was introduced into a 1.5 mL Eppendorf tube containing 1 mL of DMEM. The tubes were incubated for up to 14 days in a shaking water bath (60 agitations/minute), which was maintained at 37°C. Triplicate 200 *μ*L aliquots of the medium (containing released FITC-BSA) were withdrawn for analysis after 12 hours, 1 day, 2 days, 3 days, 4 days, 5 days, 6 days, 8 days, 10 days, 12 days, and 14 days. The temporal release of icariin was expressed as a percentage of the total amount of adsorbed icariin.

#### 2.2.4. Surgery

The calvarial critical-size bone defects in rats were established as previously described [25]. Briefly, 6 male Sprague-Dawley rats (5-week-old and weighing 180–220 g) were randomly assigned into 2 groups: either icariin-containing or non-icariin-containing SMC-PHBHHx/allogeneic BMSCs constructs. The animal care was performed in accordance with the guidelines of the Ethical Committee of Shanghai East Hospital Affiliated with Tongji University, Shanghai, China. All animal experiments were carried out according to the ethic laws and regulations of China. Critical-sized cranial defects (5 mm in diameter) were created in these rats. Briefly, the rats were anaesthetized with an intraperitoneal injection of pentobarbital (Nembutal 3.5 mg/100 g). A subcutaneous injection of 0.5 mL of 1% lidocaine as a local anesthetic was given along the sagittal midline of the skull. A sagittal incision was made over the scalp from the nasal bone to the middle sagittal crest and the periosteum was dissected. The 5 mm defects were created using a dental surgical drill with a trephine with a constant cooling rinse. Subsequently, the calvarial disk was carefully removed to avoid tearing the dura. After rinsing with physiological saline to wash out any bone fragments, samples from various groups were implanted randomly into these defects. Afterwards, the periosteum and the scalp were closed in layers with interrupted 4-0 Vicryl resorbable sutures.

#### 2.2.5. Radiographic Evaluation

Eight weeks after operation, the rats were sacrificed by intramuscular injection of overdose of Sumianxin II. All the 6 calvarial blocks of the sacrificed animals were harvested and immediately immersed into the 10% neutrally buffered formalin for fixation. Radiographic analysis of bone regeneration within the defects was performed using an X-ray unit (Vario^DG^, Sirona), with the exposure time set at 0.03 seconds. After a 2-day fixation, the specimens were scanned along the sagittal direction though by micro-CT (Inveon, Siemens) with a resolution of 18 *μ*m followed by an off-line reconstruction. After scanning, the selection of the area of interest was performed manually. Our preliminary study showed that the grey value of SMC-PHBHHx material was around −217, which was lower than water. The thresholding of mineralized bone was set at 500.

The following morphometric parameters obtained in direct mode were adopted to estimate the bone regeneration within the defects using software Siemens Inveon:

1. Relative bone volume (bone volume/tissue volume, BV/TV: %)
2. Trabecular number (Tb.N: 1/mm)
3. Trabecular separation (Tb.Sp: mm)
4. Trabecular thickness (Tb.Th: mm).

#### 2.2.6. Histomorphometric Analysis

The samples were then decalcified in 4.18% EDTA + 0.8% formalin at pH 7.2 for four weeks at 4°C, rinsed with phosphate buffer, and embedded in paraffin. Serial 6 *μ*m thickness sections were stained with hematoxylin-eosin (HE). The numbers of blood vessels were evaluated under light microscopy. The final magnification was ×50.

### 2.3. Statistical Analysis

We first used both Kolmogorov-Smirnov test and D'Agostino and Pearson omnibus normality test to comprehensively check the normality of the data of each group. According to the results, we selected either parametric tests or nonparametric tests to analyze the data. The data in concentration selection test were statically analyzed using two-way ANOVA. For the other data, we used either unpaired* t*-test or Man–Whitney test to compare the effect of icariin with the corresponding control (no icariin). The level of significance was set at *p* < 0.05. SPSS software (version 20) for a Windows computer system was employed for the statistical analysis.

## 3. Results

### 3.1. Multilineage Differentiation of Allogeneic BMSCs

**Figure 1:**
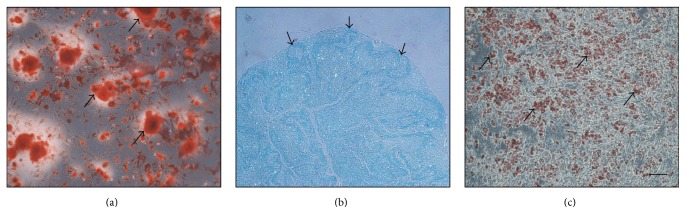
Light micrographs depicting the (a) osteogenic, (b) chondrogenic, and (c) adipogenic differentiation of multipotent BMSCs. The black arrows indicated the red-stained mineralized nodules in (a), blue-stained chondrogenic cell pellet in (b), and red-stained lipid droplets in cells in (c). Bar = 100 *μ*m.

Multilineage differentiation assay showed that the allogeneic BMSCs could differentiate into osteogenesis, chondrogenesis, and adipogenesis (Figure 1). For the osteogenic differentiation, the mineralized nodules in cell matrix—the final osteogenic differentiation marker—were stained red (indicated by black arrows in Figure 1(a)). The chondrogenic differentiation was approved by the abundant presence of glycosaminoglycans that were a typical chondrogenic differentiation marker and were stained blue in the cell pellet by alcian blue (indicated by black arrows in Figure 1(b)). The adipogenic differentiation was characterized by the oil droplets within the cells that were stained red (indicated by black arrows in Figure 1(c)).

### 3.2. Concentration Selection through Cell Proliferation and ALP Activity Assays

**Figure 2:**
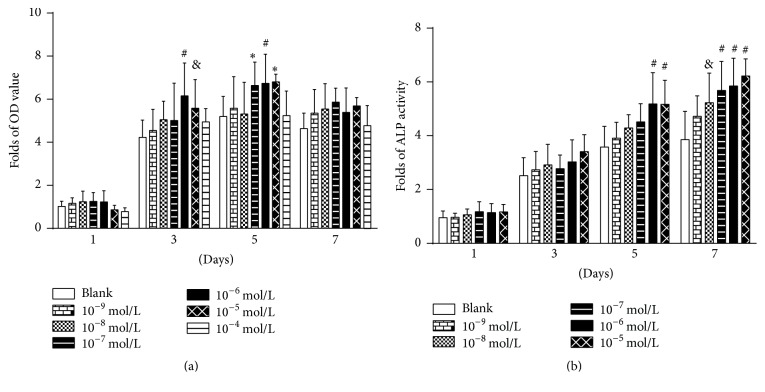
Time-course and dose-dependent tests to select the optimal concentration of icariin for inducing the osteogenic differentiation of BMSCs. Icariin of 0, 10^−9^, 10^−8^, 10^−7^, 10^−6^, 10^−5^, or 10^−4^ mol/L was used to treat preosteoblasts for 1 day, 3 days, 5 days, and 7 days. (a) Cell proliferation assays; (b) alkaline phosphatase (ALP) activity assays. All data are presented as mean values together with the standard deviation (SD). ^*∗*^ *p* < 0.05; ^&^ *p* < 0.01; ^#^ *p* < 0.001 indicating the statistical difference between the indicated group and the control group (blank) at the same time point.

Icariin at only 10^−5^ mol/L and 10^−6^ mol/L resulted in significantly higher OD value (indicator for cell proliferation) than the blank control after a 3-day treatment. On the 5th day, icariin at 10^−5^ mol/L, 10^−6^ mol/L, and 10^−7^ mol/L was associated with significantly higher OD value than the blank control. The average OD value under the induction of 10^−6^ mol/L icariin was the highest on all the selected time points (Figure 2(a)). Icariin at 10^−5^ mol/L and 10^−6^ mol/L resulted in a significantly higher ALP activity than the control after a 5-day treatment. Icariin ranging from 10^−8^ mol/L to 10^−4^ mol/L was associated with significantly higher OD value than the blank control on the 7th day. The value of ALP activity induced by 10^−6^ mol/L was the highest and second highest on the 5th day and 7th day, respectively (Figure 2(b)). Consequently, we selected 10^−6^ mol/L as the optimal concentration for icariin in the following tests.

### 3.3. Expression of Osteogenesis- and Angiogenesis-Related Genes

**Figure 3:**
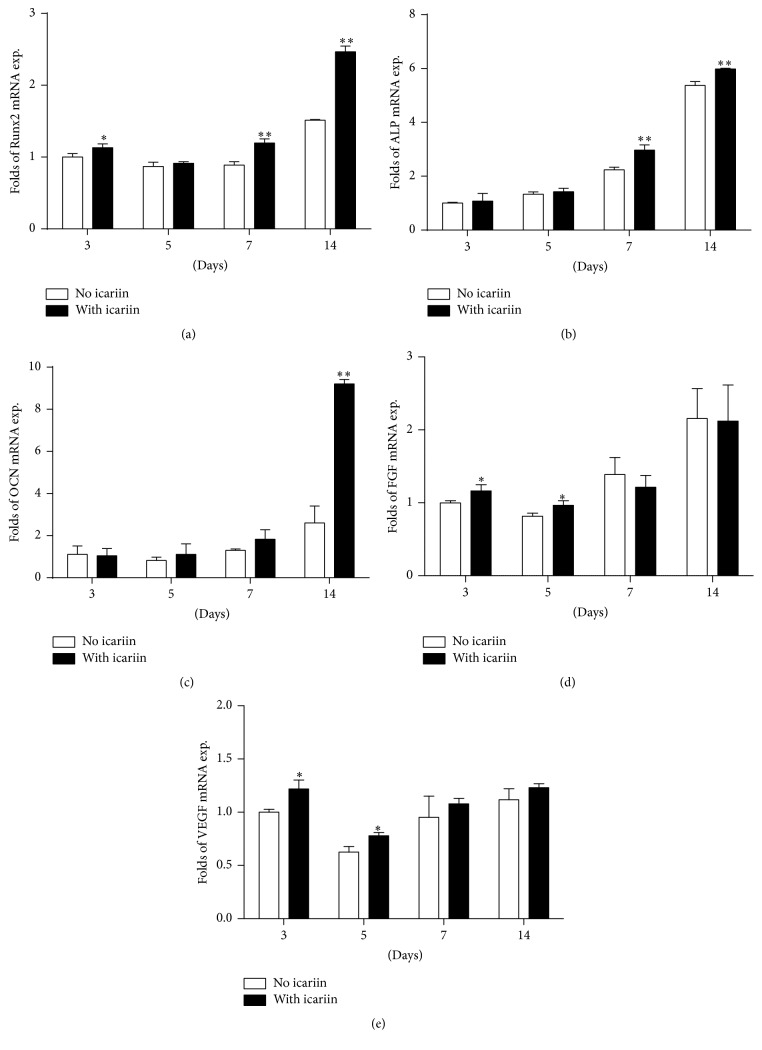
Graphs depicting the fold changes of the expression of osteogenesis-related and angiogenesis-related mRNA in BMSCs under the stimulation of icariin for 3 days, 5 days, 7 days, and 14 days. (a) Runx2, (b) alkaline phosphatase (ALP), (c) osteocalcin (OCN), (d) fibroblast growth factors (FGF), and (e) vascular endothelial growth factor (VEGF). All data are presented as mean values together with the standard deviation (SD). ^*∗*^ *p* < 0.05; ^*∗∗*^ *p* < 0.01 indicating the statistical difference between the experimental group (with icariin) and the control group (no icariin) at the same time point.

In comparison with the control (no icariin), 10^−6^ mol/L icariin could induce significantly higher expression of osteogenesis-related genes, such as Runx2 mRNA (at 3 days, 7 days, and 14 days) (Figure 3(a)), ALP mRNA (at 7 days and 14 days) (Figure 3(b)), and OCN mRNA (at 14 days) (Figure 3(c)), as well as angiogenesis-related genes, such as FGF mRNA (at 3 days and 5 days) (Figure 3(d)) and VEGF mRNA (at 3 days and 5 days) (Figure 3(e)), than the corresponding no-icariin treatments.

### 3.4. Expression of Osteogenesis- and Angiogenesis-Related Proteins

**Figure 4:**
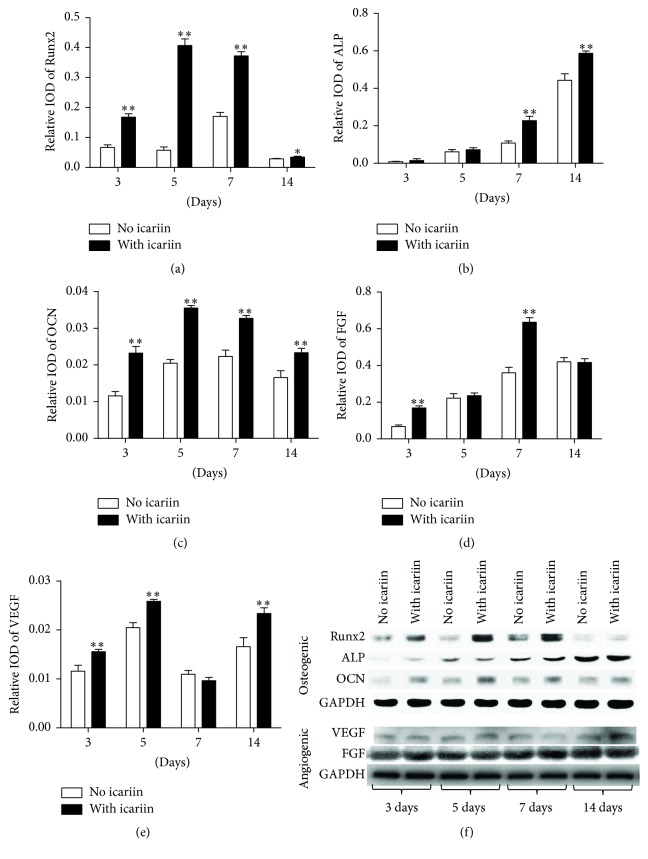
Graphs depicting the fold changes of the expression of osteogenesis-related and angiogenesis-related proteins in BMSCs under the stimulation of 10^−6^ mol/L icariin for 3 days, 5 days, 7 days, and 14 days. (a) Runx2, (b) alkaline phosphatase (ALP), (c) osteocalcin (OCN), (d) fibroblast growth factors (FGF), (e) vascular endothelial growth factor (VEGF), and (f) photographs of western blot analysis from a representative experiment. All data are presented as mean values together with the standard deviation (SD). ^*∗*^ *p* < 0.05; ^*∗∗*^ *p* < 0.01 indicating the statistical difference between the experimental group (with icariin) and the control group (no icariin) at the same time point.

Western blot analysis showed that 10^−6^ mol/L icariin could induce significantly higher expression of osteogenesis-related proteins, such as Runx2 (at all the time points) (Figure 4(a)), ALP (at 7 days and 14 days) (Figure 4(b)), and OCN (at all the time points) (Figure 4(c)), as well as angiogenesis-related genes, such as FGF (at 3 days, 5 days, and 14 days) (Figure 4(d)) and VEGF (at 3 days and 7 days) (Figure 4(e)), than the corresponding no-icariin treatments.

### 3.5. In Vitro Mineralization of Allogeneic BMSCs Induced by Icariin

**Figure 5:**
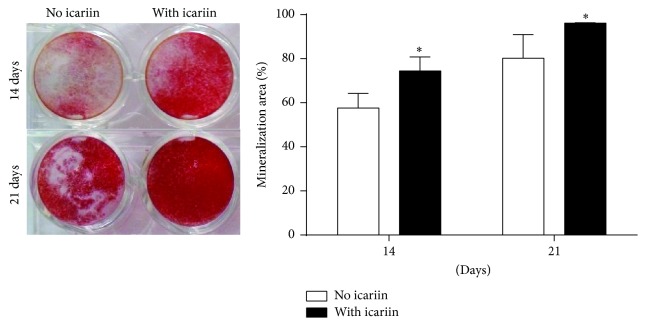
Mineralization assay of BMSCs with or without the stimulation of 10^−6^ mol/L icariin for 14 days and 21 days. All data are presented as mean values together with the standard deviation (SD). ^*∗*^ *p* < 0.05 indicating the statistical difference between the experimental group (with icariin) and the control group (no icariin) at the same time point.

The mineralization area in the group either with or without 10^−6^ mol/L icariin increased with time. On both the 14th day and the 21st day, 10^−6^ mol/L icariin resulted in significantly higher mineralization area than the control (no icariin) (Figure 5).

### 3.6. SEM Characterization of Icariin Adsorption and Cell Adhesion

**Figure 6:**
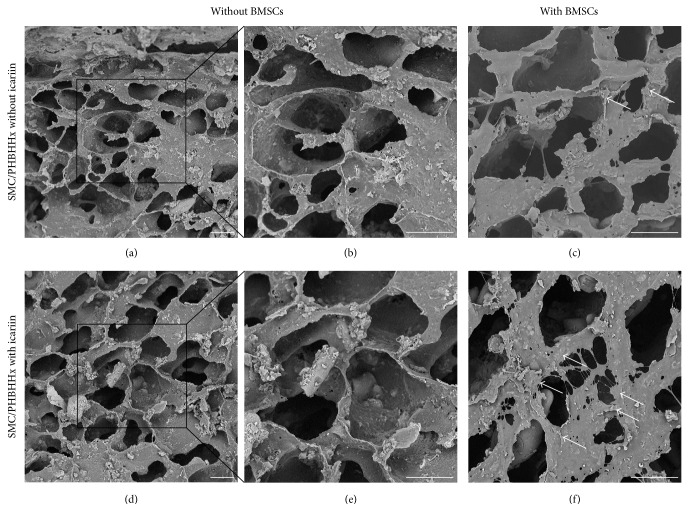
Scanning electron microscopy graphs depicting the morphology and topography of SMC-PHBHHx scaffolds without (a, b, d, e) or with (c, f) BMSCs in the absence (a, b, c) or presence (c, d, e) of 10^−6^ mol/L icariin. Bar = 30 *μ*m.

The SMC-PHBHHx composite scaffolds showed an interconnected porous structure (Figures 6(a) and 6(b)). The adsorption of icariin onto the scaffolds did not significantly change the structure and topography of the scaffold (Figures 6(c) and 6(d)). Qualitative observation showed that more BMSCs could be found on icariin-containing SMC-PHBHHx composite scaffolds than on non-icariin-containing ones (Figures 6(c) and 6(f)).

### 3.7. Release Kinetics of Icariin

**Figure 7:**
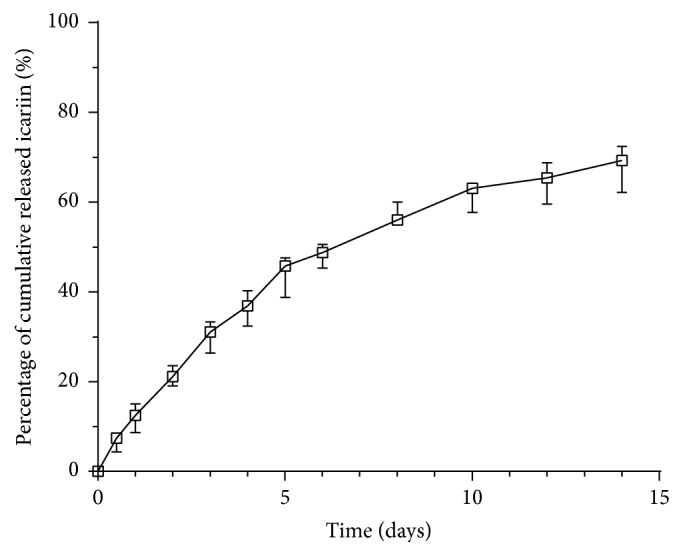
The in vitro release profile of icariin from a SMC-PHBHHx scaffold. All data are presented as mean values together with the standard deviation (SD).

The release kinetics of the adsorbed icariin from the SMC-PHBHHx composite followed a biphasic course: an initial (5 days) rapid phase and a subsequent slower phase (Figure 7). The adsorbed icariin showed a slow release profile with nearly 10% per day within the first 5 days. During the subsequent slower phase (days 5–14), the adsorbed icariin was released 2.2% per day with 70% depleted by the end of 14 days.

### 3.8. Micro-CT Analysis and Histological Observation of Newly Formed Bone Tissue

**Figure 8:**
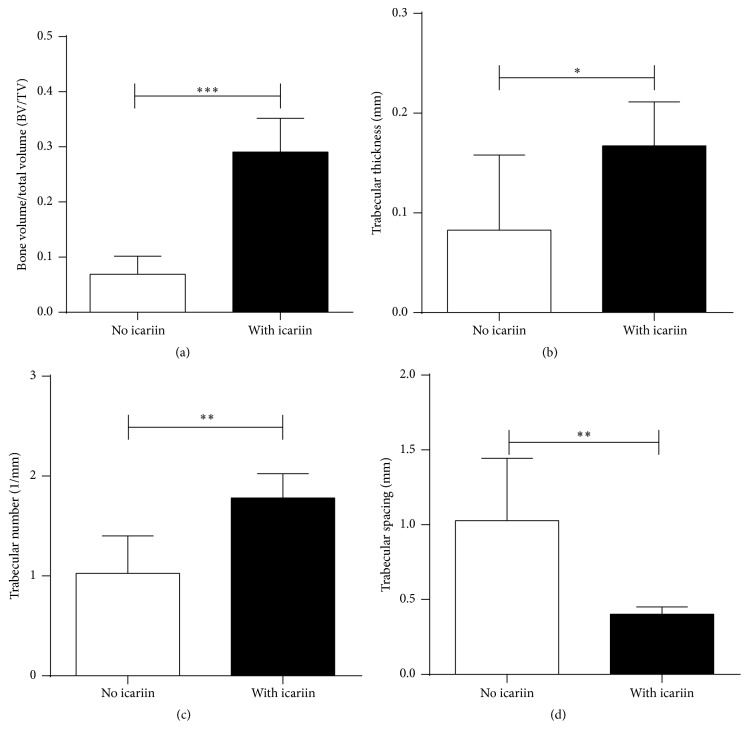
Graph depicting the micro-CT analysis of the BV/TV (a), Tb.N (b), Tb.Th (c), and Tb.Sp (d) of the newly formed bone within the calvarial critical-size bone defects that were treated with SMC-PHBHHx scaffolds/allogeneic BMSCs either without or with adsorbed icariin. All data are presented as mean values together with the standard deviation (SD). ^*∗*^ *p* < 0.05; ^*∗∗*^ *p* < 0.01; ^*∗∗∗*^ *p* < 0.001.

**Figure 9:**
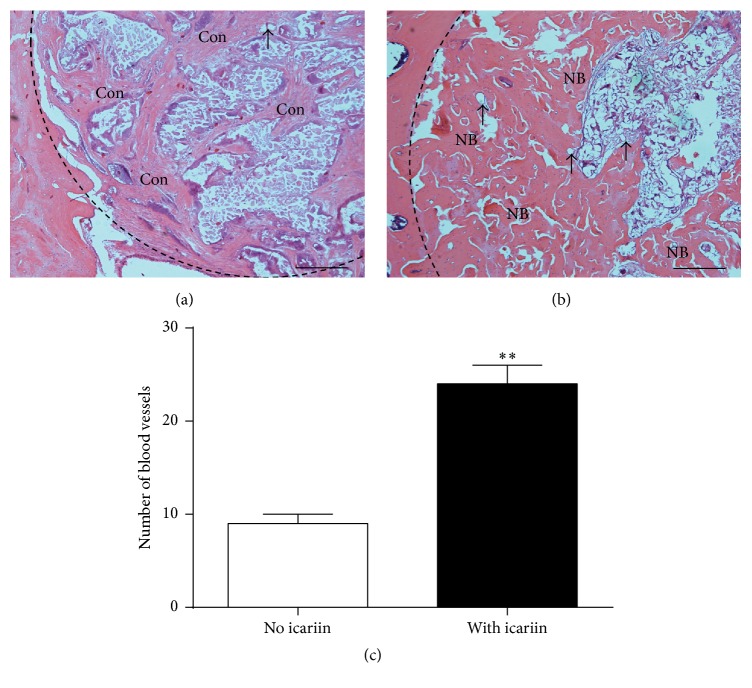
Light micrographs depicting the new bone formation within the calvarial critical-size bone defects that were treated with SMC-PHBHHx/allogeneic BMSCs either (a) without or (b) with icariin. Bar = 200 *μ*m. Then all data are presented as mean values together with the standard deviation (SD). NB: new bone; Con: connective tissue; black arrow: blood vessels. (c) Graph depicting the number of vessels per section. All data are presented as mean values together with the standard deviation (SD). ^*∗∗*^ *p* < 0.01.

Micro-CT analysis showed that the constructs with icariin resulted in significantly higher BV/TV (Figure 8(a)), Tb.Th (Figure 8(b)), and Tb.N (Figure 8(c)) and significantly lower Tb.Sp (Figure 8(d)) than the constructs with no icariin. Histological observations showed significantly less new bone formation within the defects treated with SMC-PHBHHx/allogeneic BMSCs with no icariin (Figure 9(a)) than that with icariin (Figure 9(b)).

### 3.9. Histomorphometric Analysis of Blood Vessels

Histomorphometric analysis indicated that the numbers of blood vessels within the defects treated with SMC-PHBHHx with icariin were significantly higher than those within the defects treated with SMC-PHBHHx without icariin (Figure 9(c)).

## 4. Discussion

Critical-size bone defects, a standard experimental model for large-volume bone defects, are commonly used to evaluate the treatment efficacy of novel biomaterials. The lack of osteogenic cells, osteoinductive growth factors, and osteoconductive scaffolds always leads to a nonosseous repair in a critical-size bone defect. Bone tissue engineering is a technique to integrate various knowledge in osteogenic stem cells, osteoconductive scaffolds, and osteoinductive growth factors with an aim of significantly accelerating and promoting bone regeneration. In this study, we, for the first time, showed that the slowly released icariin could significantly promote the efficacy of SMC-PHBHHx/allogeneic BMSCs for repairing critical-size bone defects.

The selection of bioactive agents is critical for the effect of a tissue engineering technique. BMPs are still the most potent growth factors for bone tissue engineering. BMPs can bind their transmembrane serine/threonine kinase receptors [26] and trigger two main downstream signaling pathways: Smad-dependent and Smad-independent signaling pathways [27]. Activated BMP receptors phosphorylate Smad1/5/8, which assembles into a complex with Smad4 and translocates to the nucleus, regulating the transcription of target genes, such as Runx2 [26]. In addition to Smad-dependent signaling, a series of Smad-independent downstream signaling pathways, including MAPK pathways, such as p38, c-Jun N-terminal kinase (JNK), and extracellular signal-related kinase (ERK), are also activated [28]. Stimulating the expression of endogenous BMPs is the pathway to exert osteoinductive effects of many drugs, such as icariin [29]. Icariin could enhance the expression of endogenous BMPs and subsequent osteogenic signaling pathways, such as Smad4, Runx2, and OPG [18, 29]. The induction of endogenous BMP-2 by icariin was, at least partially, mediated by the Wnt/*β*-Catenin-BMP signaling pathway [30]. Ohba et al. suggested two possible mechanisms for the involvement of BMP signaling in the effects of icariin [31]: (1) icariin indirectly activated BMP signaling through extracellular BMPs; (2) icariin directly activated BMP signaling by interacting with Smads via unknown mechanisms. The indispensability of endogenous BMPs for the effect of icariin was proved by the fact that noggin, an extracellular BMP antagonist, could diminish the icariin-induced enhancement of osteogenic differentiation (such as ALP, OCN, and mineralization) in osteogenic cells [18]. Consistently, the specific inhibitor for the Smad-independent ERK, JNK, and p38 MAPK signaling pathways could dramatically attenuate the promoting effect of icariin on the osteogenesis of BMSCs [32]. In addition to the BMP-associated signaling pathways, icariin could also stimulate the osteogenic differentiation of rat bone marrow stromal cells via activating the PI3K-AKT-eNOS-NO-cGMP-PKG [33]. More importantly, icariin could induce the osteogenic differentiation of BMSCs in many pathological conditions, such as osteoporosis [34, 35] and osteonecrosis [36]. Interestingly, icariin could activate different molecular cascades on BMSCs in corticosterone and ovariectomy induced osteoporotic rats [35]. Estrogen and epigenetic modulation were the newly found targets of icariin for its beneficial effect on osteogenesis in pathological conditions [34, 36]. In addition to the promoting effect on osteoblastic differentiation, icariin could also suppress osteoclastic activity, which was different from BMPs. Icariin inhibited osteoclastic differentiation in both its coculture with osteoblasts and single culture [21]. This effect was, at least partially, mediated by icariin-induced increase of OPG/RANKL expression ratios [37]. Consequently, although the potency of icariin in inducing bone formation is less than that of BMP-2, icariin is advantageous in balancing the osteoblastic and osteoclastic activity. This is especially important for the patients with osteoporosis. All these properties confer a very promising clinical application potential on icariin.

Although most of the previous reports indicated the promoting effect of icariin on osteogenesis of BMSCs, whether icariin can promote the repairing efficacy of allogeneic BMSCs in vivo is, hitherto, not known. In this study, we hypothesized that the slowly released icariin could significantly promote the efficacy of SMC-PHBHHx composite and allogeneic BMSCs for repairing critical-size bone defects. We tried to answer this question in a step-forward way. Firstly, we showed the multipotency (Figure 1) of the purchased BMSCs using well-established assays. Thereafter, we determined the optimal concentration of icariin at 10^−6^ mol/L in the time-course and dose-dependent proliferation assays (Figure 2(a)) and ALP assays (Figure 2(b)). The following RT-PCR and western blot analyses corroborated that icariin was associated with either equivalent or significantly higher level of osteogenesis-related genes and proteins, such as Runx2, ALP, and OCN (Figures 3 and 4) during the monitoring span (3–14 days). Interestingly, the icariin-induced upregulation magnitude of Runx2 and OCN proteins was much more significant than their genes on the 3rd, 5th, and 7th day, while the upregulation fold of OCN protein was much lower than OCN gene. These phenomena suggested a posttranscriptional modulation might also be involved in icariin-related effects. In the mineralization assay, we showed that 10^−6^ mol/L icariin could significantly promote the calcium nodule formation on both 14th day and 21st day (Figure 5).

A suitable scaffold is indispensable for the application of allogeneic BMSCs. In previous studies, SMC-PHBHHx composite materials showed good biocompatibility, proper stiffness, and, more importantly, the ability to carry and control release of bioactive agents [24]. Moreover, the radiolucency of this material is highly suitable for radiographic examination of new bone formation in clinic. In this study, we tried to functionalize SMC-PHBHHx with icariin in order to achieve enhanced efficacy in bone regeneration. Our data showed that the adsorbed icariin did not significantly influence the morphology of SMC-PHBHHx scaffold (Figures 6(a), 6(b), 6(d), and 6(e)). The adsorbed icariin showed a controlled slow release profile with 30% left by the end of 14 days in the in vitro condition (Figure 7). Moreover, qualitative scanning electron microscope observation showed that more BMSCs could be found on icariin-containing SMC-PHBHHx composite scaffolds than on non-icariin-containing ones (Figures 6(c) and 6(f)). These results indicated the feasibility to construct icariin/SMC-PHBHHx/allogeneic BMSCs constructs for bone tissue engineering.

Subsequently, we tested the effect of icariin on the efficacy of SMC-PHBHHx/allogeneic BMSCs in a calvarial critical-size bone defect. Eight weeks after implantation, micro-CT evaluation showed that the BV/TV, Tb.Th, and Tb.N of the new bone regenerated in the SMC-PHBHHx/allogeneic BMSCs with icariin were 4.2 times, 1.8 times, and 2.0 times higher than those in the SMC-PHBHHx/allogeneic BMSCs without icariin, respectively (Figures 8(a), 8(b), and 8(c)). The presence of icariin was also associated with a significantly lower Tb.Sp (Figure 8(d)). Consistent with the radiographic analysis, histological observation also indicated the significantly promoting effect of icariin on bone regeneration (Figures 9(a) and 9(b)). These results clearly indicated that the slowly delivered icariin could promote bone regeneration of SMC-PHBHHx/allogeneic BMSCs in critical-size bone defects.

In addition to the direct promoting effect, icariin may also benefit bone regeneration through enhancing angiogenesis. Vascularization is a crucial step in bone regeneration, which brings mesenchymal stem cells and nutrition to wounds [38]. Icariin could promote not only in vitro endothelial tubulogenesis assay but also in vivo angiogenesis [39], possibly through activating EGF-EGFR pathway and thereafter endothelial NO synthase [40]. Moreover, icariin could directly stimulate angiogenesis through activating a series of angiogenic signals, such as ERK, PI3K, and Akt [41]. In our in vitro cellular experiments, icariin could significantly promote the angiogenesis-related genes and proteins, such as VEGF and FGF (Figures 3 and 4). Accordingly, our histomorphometric analysis indicated that the slowly released icariin resulted in significantly higher number of blood vessels (Figure 9(c)).

This study bears also some limitations, such as the limited group setup in the animal studies and no in vivo tracking of allogeneic BMSCs. Consequently, the contribution of allogeneic and autologous BMSCs could not be precisely determined. However, our findings clearly showed that the slowly delivered icariin could promote the efficacy of SMC-PHBHHx/allogeneic BMSCs for healing the critical-bone defects in rats. Such an effect may be mediated by icariin-induced upregulation of osteogenesis and angiogenesis. With the indication of the current study, we are trying to further explore these factors.

## Figures and Tables

**Table 1:** Primer sequences for real-time quantitative polymerase chain reaction analysis of the expression of osteogenic genes (Runx2, alkaline phosphatase (ALP), and osteocalcin (OCN)) and angiogenic genes (vascular endothelial growth factor (VEGF) and fibroblast growth factors (FGF)).

Gene	Primers (F = forward; R = reverse)
ALP	F: 5′-GTC CCA CAA GAG CCC ACA AT-3′; R: 5′-CAA CGG CAG AGC CAG GAA T-3′
OCN	F: 5′-CAG TAA GGT GGT GAA TAG ACT CCG-3′; R: 5′-GGT GCC ATA GAT GCG CTT G-3′
Runx2	F: 5′-TCT TCC CAA AGC CAG AGC G-3′; R: 5′-TGC CAT TCG AGG TGG TCG-3′
VEGF	F: 5′-CTT GAG TTG GGA GGA GGA TG-3′; R: 5′-TGG CAG GCA AAC AGA CTT C-3′
FGF	F: 5′-CTC TGT CTC CCG CAC CCT AT-3′; R: 5′-CCT TCC ACC CAA AGC AGT AG-3′
GAPDH	F: 5′-GGC AAG TTC AAC GGC ACA GT-3′; R: 5′-GCC AGT AGA CTC CAC GAC AT-3′
